# Editorial: Change in epidemiology and etiology of respiratory tract and gastrointestinal infections during COVID-19 pandemic

**DOI:** 10.3389/fmicb.2024.1473567

**Published:** 2024-08-15

**Authors:** Van Thuan Hoang, Philippe Gautret, Jaffar A. Al-Tawfiq

**Affiliations:** ^1^Thai Binh University of Medicine and Pharmacy, Thai Binh, Vietnam; ^2^IHU-Méditerranée Infection, Marseille, France; ^3^Aix Marseille Univ, AP-HM, SSA, RITMES, Marseille, France; ^4^Specialty Internal Medicine and Quality Patient Safety Department, Johns Hopkins Aramco Healthcare, Dhahran, Saudi Arabia; ^5^Infectious Diseases Division, Department of Medicine, Indiana University School of Medicine, Indianapolis, IN, United States; ^6^Infectious Diseases Division, Department of Medicine, Johns Hopkins University School of Medicine, Baltimore, MD, United States

**Keywords:** COVID-19, SARS-CoV-2, epidemiology, respiratory tract infections, gastrointestinal infections

The COVID-19 pandemic has profoundly impacted global health, not only through the direct effects of SARS-CoV-2 but also by altering the landscape related to other respiratory and gastrointestinal infections. As researchers have focused some attention to the understanding of these shifts, our Research Topic, “*Change in Epidemiology and Etiology of Respiratory Tract and Gastrointestinal Infections during COVID-19 Pandemic*,” has gathered a diverse Research Topic of studies that shed light on these changes. This editorial aims to frame the significance of such research, to summarize key findings from the contributing articles, and to contextualize their implications for future public health strategies.

The COVID-19 pandemic imposed unprecedented public health measures at country scales, such as social distancing, lockdowns, and enhanced hygiene practices (Han et al., 2020). These measures not only influenced the transmission dynamic of SARS-CoV-2 but also had consequential effects on other infectious diseases (Kaba et al., 2021; Chow et al., 2023). Eight original articles (Pan et al.; Cao et al.; Zhou et al.; Guo et al.; Yokoyama et al.; Korsun et al.; Alemayehu et al.; Zhu et al.) and one review (He et al.) in this Research Topic explore these phenomena from various angles, providing valuable insights into the complex interplay between the pandemic and other pathogens.

Pan et al. offered a comprehensive analysis of how COVID-19 control measures impacted the prevalence of other respiratory viruses among 2,744 patients with acute respiratory tract infections in Shanghai, China. The authors found a significant reduction in the detection of common respiratory viruses, highlighting the collateral benefits of COVID-19 mitigation strategies on respiratory infection. Indeed, at the beginning of the pandemic, there were no effective vaccines available globally, hence non-pharmaceutical interventions (NPIs) such as social distancing, wearing masks, practicing hand hygiene, and delaying the spring 2020 schools' semester were implemented to prevent the spread of SARS-CoV-2 (Li et al., 2022). These interventions impacted other respiratory viruses, including influenza, which was the most prevalent viral pathogen among the respiratory pathogens (Ye et al., 2017).

The COVID-19 pandemic particularly influenced the evolutionary dynamics of respiratory viruses (Chow et al., 2023). Guo et al. provided a comparative analysis of respiratory syncytial virus (RSV) genetic diversity before and during the pandemic using sequences collected from the NCBI GenBank Database. The study indicated shifts in evolutionary pressures, but no substitutions that altered the structural conformations of the antigenic sites. These findings suggested that the intensive NPIs during the COVID-19 pandemic did not influence the evolutionary patterns of RSV. The incidence of RSV after the main part of the pandemic was analyzed in another study conducted by Korsun et al. in Bulgaria. A considerable resurgence of this virus was reported during autumn 2022, following the lifting of NPIs for COVID-19.

In addition, Zhu et al. highlighted the re-emergence of *Mycoplasma pneumoniae* as COVID-19 restrictions were eased in Shanghai, China. Although these outbreaks may represent a return to the cyclical respiratory pathogens similar to the time before the COVID-19 pandemic (Upadhyay and Singh, 2024). They underscore the need for continuous vigilance and adaptability in public health responses to prevent and control outbreaks of other infectious diseases.

Another path to try understanding the COVID-19 impact was to determine the factors that influenced viral clearance. Cao et al. delved into the role of immune markers like IgG and IL-6 in predicting the duration of viral shedding. Their findings showed that age, IgG, and IL6 could potentially serve as useful predictors for SARS-CoV-2 RNA clearance exceeding 14 days in asymptomatic and mild COVID-19 patients in Tianjin, China. This information can assist in estimating which patients might have a shorter recovery period, offering valuable insights for clinical prevention and control strategies. In fact, achieving rapid clearance of SARS-CoV-2 during the early stages of infection is desirable, as it can help prevent viral spread within the body and minimize tissue damage and severe clinical outcome (Adhikari et al., 2020).

The COVID-19 pandemic also complicated the management of secondary infections. Zhou et al. provided a critical examination of the challenges faced by healthcare providers in managing secondary infections, and co-infections among vulnerable populations in Beijing, China. These authors found that predictors of secondary pulmonary infection and co-infection were as follow: severe COVID-19 disease, ICU admission within 48 h of hospitalization, PCT >0.5 ng/ml. and cerebrovascular diseases. Their study emphasized the importance of integrated care approaches and robust infection control practices in mitigating the impact of secondary infections. As they found that increased PCT was a strong prognostic factor, elevated PCT level could indicate antibiotic treatment need in these cases (Almulhim et al., 2024).

During the initial phase of COVID-19, patients often experience gastrointestinal symptoms (Mao et al., 2020). The severity of COVID-19 has been linked to changes in gut microbiota, with more pronounced dysbiosis observed in patients with more severe illness (Neag et al., 2022). This is thought to be due to the high expression of ACE2, which serves as the entry point for SARS-CoV-2 into the gastrointestinal system (Ni et al., 2020). Moreover, different SARS-CoV-2 variants might alter the gut microbiota and metabolites. This was illustrated in a Japanese study by Yokoyama et al. in this Research Topic. Fecal microbiome analysis showed that α-diversity was reduced in the order of the Omicron, Delta, and Alpha variants and that the Omicron and that Delta variants had markedly reduced propionic and lactic acid levels compared to the Alpha strain (*p* < 0.05). Other studies on the gut microbiota of COVID-19 patients reported that the abundance of some bacteria, such as *Faecalibacterium prausnitzii, Eubacterium rectale*, and various *Bifidobacteria* species, decreased as the disease progressed (Yeoh et al., 2021; Zhang et al., 2023). Additionally, metabolomic studies indicated a reduction in short-chain fatty acids and L-isoleucine production in the gut microbiota, which was linked to the severity of the disease (Zhang et al., 2023).

A narrative review conducted by He et al. explored the persistence and management of gastrointestinal symptoms in long-term COVID-19 patients. Such symptoms are caused by factors such as SARS-CoV-2 infection of intestinal epithelial cells, cytokine storm, gut dysbiosis, therapeutic drugs, psychological factors, and the worsening of pre-existing conditions. Interventions like probiotics, prebiotics, fecal microbiota transplantation, and antibiotics were shown to help maintaining the intestinal microecological balance and reducing gastrointestinal symptoms. This study's findings highlight the need for ongoing research into the mechanisms and treatments for such persistent symptoms.

Finally, in a study of Alemayehu et al. conducted in Ethiopia, a direct impact of NPIs against COVID-19, especially interventions in water, sanitation, and hygiene, was shown to significantly reduce the incidence of childhood diarrhea, suggesting that some pandemic-era practices could be beneficial if continued post-pandemic.

The collective findings of these studies underscore the multifaceted impact of the COVID-19 pandemic on respiratory and gastrointestinal infections. While the pandemic was associated with numerous challenges, it also provides that helped to understand the dynamics of other infectious diseases in unprecedented ways. As we move forward, the insights gained from this Research Topic will be valuable in shaping more effective public health strategies, enhancing our preparedness for future pandemics, and improving the overall management of respiratory and gastrointestinal infections. We extend our gratitude to all the contributing authors for their valuable research and to the reviewers for their insightful feedback. Together, these contributions have significantly advanced our understanding of the intricate interplay between COVID-19 and other infectious diseases.
